# Validity evidence for flourishing as a measure of global wellbeing: a national multicenter study of academic general surgery residents

**DOI:** 10.1007/s44186-022-00008-1

**Published:** 2022-02-11

**Authors:** Carter C. Lebares, Anya L. Greenberg, Paul A. Gonzales, Christy K. Boscardin, and the General Surgery Research Collaborative on Resident Wellbeing

**Affiliations:** 1 Department of Surgery, University of California San Francisco, 513 Parnassus Avenue, HSW 1601, 0790, San Francisco, CA 94143 USA; 2 Departments of Medicine and Anesthesia, Univeristy of Carlifornia San Francisco, 513 Parnassus Avenue, HSW 1601, 0790, San Francisco, CA 94143 USA

**Keywords:** Flourishing, Graduate medical education, General surgery, Resident wellbeing

## Abstract

**Purpose:**

Physician wellbeing is critical to high-quality sustainable healthcare and optimal patient experience. Few objective measures exist to assay wellbeing (as opposed to just pathology) in surgery, or to evaluate the efficacy of wellbeing interventions. Flourishing (as measured by the Mental Health Continuum, MHC) has been suggested as a concise measure of global wellbeing in surgeons. We aimed to establish validity evidence for flourishing in a large national sample of surgical trainees, explore differences by gender and race, and confirm support for the underlying constructs.

**Methods:**

This cross-sectional study of all General Surgery residents at 16 ACGME-accredited academic programs included an online survey of published measures distributed in February 2021. The Mental Health Continuum (MHC), a three-factor model, assesses emotional, social, and psychological wellbeing and is an established metric of psychosocial thriving in non-physicians. A global score cut-off exists for flourishing which represents high wellbeing. Correlation between flourishing and established measures of risk and resilience in surgery were assessed for validity evidence. Differences by gender and race were explored. A confirmatory factor analysis (CFA) was performed to confirm the three-factor structure in surgical trainees.

**Results:**

300 residents (60% non-male, 41% non-white) responded to the survey. For the overall group, flourishing was significantly *positively* correlated with all wellbeing resilience factors and *negatively* correlated with all risk factors. This held true for race and gender subgroups based on interaction analyses. CFA and sensitivity analysis results supported the three-factor structure.

**Conclusions:**

Our findings offer validity evidence for flourishing as a measure of global wellbeing and confirm the three-factor structure of emotional, social, and psychological wellbeing in surgical trainees. Thus, the MHC may be a concise tool for assaying wellbeing, within and across subgroups, and for assessing wellbeing intervention effectiveness within the surgery.

## Background

A critical balance exists between the inherent physical, psychological, and existential challenges of surgery and each surgeon’s reservoir of wellbeing [10, 25]. As a profession, surgery has the potential to deliver deep satisfaction and a sense of realized purpose. Yet, endless non-medical administrative responsibilities, the erosion of work-life boundaries, and an increasingly industrialized approach to healthcare threaten to overwhelm the rewards of this profession [77]. As a result, surgeons are at risk for pathology such as burnout and post-traumatic stress disorder [29, 41, 44], and patients are at risk of suboptimal care or loss of the precious physician–patient bond. International consensus recognizes that safe sustainable healthcare, best outcomes for patients, and the maintenance of ethical standards in medicine depend on prioritizing workplace systems and culture that promote physician wellbeing [6, 8, 10, 42, 43, 67].

However, due to the lack of tools for assessing global wellbeing in surgeons, a critical gap in our understanding remains. Without an established measure of wellbeing (one that accounts for individual differences and the modern conceptualization of wellbeing as a complex state of positive affect, belonging, and purpose), [18, 21–23, 64], our ability to evaluate individual wellbeing status, assess intervention efficacy, and target precious resources is limited.

In non-physicians, flourishing (as measured by the Mental Health Continuum-Short Form, MHC-SF; for simplicity referred to herein as MHC) is an established metric of clinically-relevant psychosocial thriving defined as emotional, social, and psychological wellbeing. Grounded in theory and empirical evidence, the MHC is widely regarded as an assay of ‘fulfillment’ (as opposed to more fleeting ‘happiness’), reflecting the conceptualization of wellbeing as a state of positive emotions, positive relationships, and a healthy sense of community [19, 34, 36, 61, 63]. In our prior work [42, 43], using a cross-sectional single institution survey, we showed a correlation between flourishing and multiple established wellbeing risk and resilience factors in General Surgery residents, suggesting flourishing may be an accurate metric of psychosocial thriving in surgeons. Further, we found flourishing to be influenced by the (inherent) mindfulness tendencies of residents and by elements of workplace support, suggesting it may be sensitive to certain individual and workplace-level interventions. In a subsequent longitudinal cohort study of mixed-specialty postgraduate year (PGY)-1 trainees who received a tailored mindfulness-based intervention called Enhanced Stress Resilience Training (ESRT) [45], we found flourishing to be significantly positively associated with higher individual mindfulness, higher perceived workplace support, and lower negative emotions (e.g., stress, anxiety), both cross-sectionally and longitudinally. These preliminary studies reinforced our line of reasoning that flourishing may be a measure of global wellbeing in surgical trainees and that modifiable individual and workplace factors exist which may influence flourishing (and, therefore, trainee wellbeing). A large body of research in both behavioral psychology and occupational science shows that individual cognitive training [8, 24], and the provision of workplace support can mitigate workplace stress, increase job satisfaction, and improve clinician wellbeing overall [2, 77, 79]. Nonetheless, knowledge gaps remain that our single-center focus and small sample size could not address.

In the current national study of academic General Surgery programs, we aimed to establish validity evidence for flourishing as a measure of global (i.e., multi-faceted) wellbeing in surgical trainees, exploring differences by gender and race. We hypothesized flourishing would be associated with the presence of factors shown in surgery to influence resilience [37, 42, 43, 45, 57, 58, 79] (i.e., “resilience factors”, like mindfulness, personal accomplishment, and workplace social support) and distress (i.e., “risk factors”, like burnout, job dissatisfaction, and depression) [30, 44, 65, 66, 79]. We further aimed to evaluate the three-factor structure of the MHC using a confirmatory factor analysis (CFA). Thus, the MHC and flourishing may provide metrics for the selection of high impact targets in wellbeing research, measures to assess intervention efficacy in real time, and the means to evaluate individual surgeon wellbeing in a comprehensive and efficient fashion.

## Methods

### Study design

An online survey instrument was distributed in January 2021 to all preliminary and categorical General Surgery residents (both clinically active and in research) at 16 ACGME-accredited academic training programs representing Western, Mountain, Central and Eastern regions of the US, ranging in size from 21 to 108 residents. Participating programs comprise the General Surgery Research Collaborative on Resident Wellbeing, which evolved during the first surge of Coronavirus Disease 2019 due to outreach from the UCSF Center for Mindfulness in Surgery. Champions at participating programs disseminated the survey to their respective resident bodies. 891 residents across the 16 programs received the survey, which remained open for six weeks. The study was approved by UCSF’s institutional review board and informed consent was obtained for all participants.

### Survey instrument

The anonymous survey collected basic demographic information and measured the presence of resilience, which is characterized by high positive emotions, nonreactivity to stressors, and connectedness (as defined by seminal works in the field of resilience science [18, 23, 50, 51, 59, 69] and used in prior studies as proxy measures of wellbeing [10, 42, 42, 43, 43, 44, 46, 53]). The survey further measured distress, characterized by high burnout, stress, anxiety or depressive symptoms (as defined by multiple works exploring distress in surgery and perceived in the literature to be discordant with wellbeing [28, 44, 46]. These Likert scale-based measures, found reliable in our prior work with surgical trainees, were scored according to published methods described hereinafter.

Our primary outcome variable, flourishing, was assessed through the *Mental Health Continuum-Short Form (MHC-SF)*, a 14-item measure of psychosocial wellbeing with a three-factor model reflecting social, emotional, and psychological mental health domains, with high internal consistency (> 0.80) [33] and supporting literature base of clinical relevance [25]. Similar to standard diagnostic criteria for depression, the MHC-SF items are scored according to the frequency with which respondents experience each symptom of positive mental health. Per convention, categorical designation using this measure is not limited to a specific numeric cut-off. Rather, flourishing represents experiencing high positive functioning and high positive emotions ‘every day’ or ‘almost every day.’ Scores can also be treated continuously [40]. In our work, we use both the categorical (i.e., ‘flourishing’) and continuous (i.e., ‘MHC score’) forms.

To assess individual-level risk and resilience factors, we used several published, widely accepted measures from our prior work. The *Cognitive Affective Mindfulness Scale-Revised (CAMS-R)* is a 10-item measure of both dispositional and trained mindfulness in the form of attention, present-focus, awareness, and acceptance, with internal consistency (0.7–0.74) [20] and a calculated global score sensitive to mindfulness training [25]. Higher CAMS-R scores are associated with lower odds of distress in surgical trainees [20, 44]. The *abbreviated Maslach Burnout Inventory (MBI)* is a 9-item validated screen [48] for high emotional exhaustion, depersonalization, and personal accomplishment (use and scoring described by McManus et al. [52]) associated with multiple negative sequelae in surgical trainees [17, 37, 42–44]. *Cohen’s Perceived Stress Scale (PSS)* is a 10-item widely-used measure of stress, with high internal consistency (> 0.80) [12, 54] with normative data for men and women aged 18–34. High PSS scores correlate with cognitive impairment, missed work and disability [55]. *Spielberger’s State Trait Anxiety Index (STAI)* is a 6-item measure of subjective feelings (e.g., apprehension, tension) and autonomic arousal [1, 26, 39, 71, 73] correlated with state anxiety. A cutoff of ≥ 40 was used in other studies to denote high anxiety [1, 39]. In surgical trainees, during real-life and simulated trauma scenarios the STAI has high internal consistency (0.92) ([72]. The *Patient Health Questionnaire-8 (PHQ-8)* is an 8-item rigorously evaluated and validated depression screening tool [15] with high internal consistency (0.88) [68]. A total score of > / = 10 is correlated with increased use of clinical resources [63].

Finally, we explored the influence of risk and resilience factors within the workplace through the *Swedish Demand-Control-Support Questionnaire (DCSQ)*, which is a 16-item measure of job strain with good internal consistency (0.7–0.85) [57, 58] rooted in Job Demand-Resource theory, with subdomains for Demand, Control and Support. High workplace demand and low control are known risks for job strain, while high workplace control and social support are shown to decrease risk and mitigate the effects of demand [13, 57, 58]. High subdomain designations are defined as scores within the upper third of the total possible score [57, 58].

### Data analysis

Survey respondents of all PGY-years (including research residents) were included in our data analysis given our aim to establish validity evidence for flourishing as a measure of global wellbeing in residents undergoing surgical training at academic programs, which by default include residents undertaking research years*.* Correlation between the binary flourishing variable and each risk and resilience factor was assessed using logistic regression. Correlation between total MHC score and each risk and resilience factor was assessed using linear regression. Unadjusted logistic regression models for flourishing vs. all risk and resilience factors and unadjusted linear regression models for MHC score vs. all risk and resilience factors with gender and race interaction were performed. Hypothesis tests were two-sided, and the significance threshold was set to 0.05. Statistical analyses were performed using SAS version 9.4.

To evaluate the three-factor structure of the MHC in surgical trainees, confirmatory factor analysis (CFA) of the MHC’s three factors (emotional, social, and psychological wellbeing) was conducted using a Full Information Maximum Likelihood (FIML) method of the CALIS procedure in SAS. CFA is a hypothesis testing analytic technique belonging to the structural equation modeling framework. It examines the covariance structure of variables to “confirm” (verify) the hypothesized relationship using a smaller number of unobserved (latent) variables called factors. We hypothesized that the three-factor model would be supported, verifying that the MHC measures social, emotional, and psychological mental health domains. Demonstrating the three-factor structure in surgical trainees would allow relationships between latent factors and other concepts (such as the risk of developing suicidality or workplace absenteeism) to be reliably compared across groups, allowing us to attribute differences in scores to the groups themselves, not to the items functioning differently.

To assess the adequacy of the model, we examined multiple indices, rather than a single fit index [4, 7, 11, 76], including Bentler’s Comparative Fit Index (CFI) [3], the maximum likelihood (ML)-based standardized root mean residual (SRMR), and root mean square error of approximation (RMSEA) [9, 74]. CFI values of at least 0.9 and SRMR values less than 0.08 were used to indicate acceptable fit, by convention using 0.6 as the cut-off for acceptable loading [27]. Values of 0.05 or less on the RMSEA were used to indicate a close fit, values above 0.05 and below 0.1 indicate adequate fit, and values greater than 0.1 are unacceptable. Standardized loading estimates and subscale correlations were calculated and were used together with information from the multiple fit indices, to determine the wellness of fit [11]. A sensitivity CFA was also performed, removing two items from the model with the lowest loading estimates.

## Results

### Respondents

300 residents (60% non-male, 41% non-white) responded to the survey, representing a 34% response rate (Table 1). While the distribution of our respondents by PGY-level is on par with the demographics of the entire body of US General Surgery residents [17, 81], it is slightly skewed toward non-male and non-white residents.

**Table 1:** Participant characteristics of 300 survey respondents

Characteristic	No. (%)
Self-reported gender identity	
Non-Male	179 (59.6)
Female	178 (59.3)
Genderqueer / Gender nonconforming	1 (0.3)
Transgender man	0 (0.0)
Transgender woman	0 (0.0)
Male	119 (39.7)
Decline to state	2 (0.7)
Self-reported race/ethnicity	
White	171 (57.0)
Non-White	123 (41.0)
Asian (includes Asian + White)	70 (23.3)
Latinx (includes Latinx + White)	28 (9.3)
Black/African American (includes Black/African American + White)	11 (3.7)
Other	11 (3.7)
American Indian/Alaska Native (includes American Indian/Alaska Native + White)	3 (1.0)
Native Hawaiian or Other Pacific Islander	0 (0)
Unknown/Decline to state	6 (2.0)
Training level	
Junior residents	171 (57.0)
PGY-1	77 (25.7)
PGY-2	42 (14.0)
PGY-3	52 (17.3)
Research residents	52 (17.3)
Senior residents	75 (25.0)
PGY-4	43 (14.3)
PGY-5	32 (10.7)
Decline to state	2 (0.7)

### Validity evidence for flourishing as a measure of wellbeing in surgical trainees

Both flourishing as defined by MHC cut-off and total MHC score were significantly positively correlated with all measured resilience factors (high mindfulness, personal accomplishment, workplace support and perceived workplace control) and significantly negatively correlated with all measured risk factors (high depression, emotional exhaustion, depersonalization, stress, anxiety, and perceived workplace demand) (Table 2).

**Table 2:** Associations between established resilience/risk factors and flourishing/MHC scores

Independent variable	Dependent variable					
	Flourishing (Dichotomization of MHC score)			MHC score		
	OR	95% CI	*p* value	*Β*	SE	*p* value
Resilience						
CAMS-R (Mindfulness)	1.24	1.17–1.32	< 0.001	1.42	0.13	< 0.001
MBI-PA (Personal Accomplishment)	1.54	1.36–1.76	< 0.001	3.07	0.24	< 0.001
DCSQ-Support (Workplace Support)	1.42	1.28–1.58	< 0.001	2.44	0.21	< 0.001
DCSQ-Control (Workplace Control)	1.43	1.25–1.63	< 0.001	2.29	0.35	< 0.001
Risk						
PHQ (Depressive Symptoms)	0.72	0.66–0.78	< 0.001	− 1.88	0.12	< 0.001
MBI-EE (Emotional Exhaustion)	0.77	0.71–0.84	< 0.001	− 1.67	0.17	< 0.001
MBI-DP (Depersonalization)	0.86	0.81–0.91	< 0.001	− 1.08	0.18	< 0.001
PSS (Stress)	0.76	0.71–0.81	< 0.001	− 1.48	0.09	< 0.001
STAI (Anxiety)	0.69	0.62–0.76	< 0.001	− 2.28	0.17	< 0.001
DCSQ-Demand (Workplace Demand)	0.83	0.74–0.93	< 0.001	− 1.42	0.34	< 0.001

*MHC* Mental Health Continuum, *CAMS-R*  Cognitive and Affective Mindfulness Scale-Revised, *MBI-PA*  Maslach Burnout Inventory-Personal Accomplishment, *DCSQ-Support* Demand, Control, Support Questionnaire-Support, *DCSQ-Control*  Demand, Control, Support Questionnaire-Control, *PHQ* Patient Health questionnaire, *MBI-EE* Maslach Burnout Inventory-Emotional Exhaustion, *MBI-DP* Maslach Burnout Inventory-Depersonalization, *PSS* Perceived Stress Scale, *STAI* State-Trait Anxiety Index, *DCSQ-Demand* Demand, Control, Support Questionnaire-Demand, *OR* Odds Radio, *CI*  Confidence Interval

### Validity evidence for flourishing as a measure of wellbeing by race and gender

Our sample was not powered to perform subgroup analyses. Thus, to establish validity evidence for flourishing as a measure of global wellbeing across gender and race subgroups, we performed interaction analyses exploring for differences in the influence of risk and resilience factors on flourishing/MHC score by gender-identity and race. DCSQ-control was positively associated with MHC score for males and non-males, but the effect was significantly greater for males with a 1.36 greater increase in MHC score per each unit increase in DCSQ-control (interaction estimate = 1.36, SE = 0.68, *p* = 0.05). Interaction analysis did not reveal any other significantly different effects in terms of gender or race.

### Confirmatory factor analysis

The sample size was sufficient for the CFA [5, 38, 62]. Overall determination of model fit was based on three methods: model fit indices, covariance structure, and factor correlations representing internal consistency. The model had the following fit index values: CFI = 0.89, SRMR = 0.066, RMSEA = 0.126. By conventional standards, CFI and the RMSEA each indicate an inadequate fit (CFI < 0.9, RMSEA > 0.1), but the SRMR indicates acceptable fit (< 0.08) representing a lack of consensus. Table 3 presents the covariance structure analysis, the standardized factor loading estimates, and standard errors for each item. All factor loadings are statistically significant (*p* < 0.0001), indicating that the prescribed relationships between each item and its allotted factor exist, and all loading estimates are greater than 0.6. In addition, all factors are significantly correlated with each other (*p* < 0.001 for correlation), as expected. The covariance structure analysis and factor correlations (Table 3) largely support the proposed model, overall confirming the three-factor structure.

**Table 3:** Covariance structure analysis

**MHC Item** *During the past month how often did you feel…*	Standardized factor loading (standard error)*		
	Emotional wellbeing	Social wellbeing	Psychological wellbeing
**MHC 1** …Happy	0.88 (0.02)		
**MHC 2** …Interested in life	0.89 (0.02)		
**MHC 3** …Satisfied with life	0.91 (0.01)		
**MHC 4** …That you had something important to contribute to society		0.81 (0.02)	
**MHC 5** …That you belonged to a community (like a social group, school, neighborhood)		0.78 (0.03)	
**MHC 6** …That our society is a good place, or is becoming a better place, for all people		0.68 (0.04)	
**MHC 7** …That people are basically good		0.62 (0.04)	
**MHC 8** …That the way our society works made sense to you		0.65 (0.04)	
**MHC 9** …That you liked most parts of your personality			0.84 (0.02)
**MHC 10** …Good at managing the responsibilities of your daily life			0.76 (0.03)
**MHC 11** …That you had warm and trusting relationships with others			0.81 (0.02)
**MHC 12** …That you had experiences that challenged you to grow and become a better person			0.78 (0.02)
**MHC 13** …Confident to express your own ideas and opinions			0.81 (0.02)
**MHC 14** …That your life has a sense of direction or meaning to it			0.86 (0.02)

*MHC* Mental Health Continuum

*All factor loadings are statistically significant (*p* < 0.0001), and all factors are significantly correlated with each other (*p* < 0.001)

### Sensitivity analysis

A sensitivity analysis was conducted to determine the impact of removing two items from the model with the lowest loading estimates, both from the construct subdomain of social wellbeing (MHC7 ‘During the past month, how often did you feel…that people are basically good?’, estimate 0.617, and MHC8 ‘…that the way our society works made sense to you?’, estimate 0.648). Removal of these two items produced similar results but with improved fit index values (CFI = 0.97, SRMR = 0.032, RMSEA = 0.077), making the SRMR and RMSEA both acceptable (< 0.08) and the CFI fit good (> 0.95). Table 4 presents standardized factor loading estimates from the sensitivity analysis model.

**Table 4:** Covariance structure analysis following sensitivity analysis

**MHC Item** *During the past month how often did you feel…*	Standardized factor loading (standard error)*		
	Emotional wellbeing	Social wellbeing	Psychological wellbeing
**MHC 1** …Happy	0.88 (0.02)		
**MHC 2** …Interested in life	0.88 (0.02)		
**MHC 3** …Satisfied with life	0.91 (0.01)		
**MHC 4** …That you had something important to contribute to society		0.84 (0.02)	
**MHC 5** …That you belonged to a community (like a social group, school, neighborhood)		0.78 (0.03)	
**MHC 6** …That our society is a good place, or is becoming a better place, for all people		0.58 (0.04)	
**MHC 7** …That people are basically good			
**MHC 8** …That the way our society works made sense to you			
**MHC 9** …That you liked most parts of your personality			0.84 (0.02)
**MHC 10** …Good at managing the responsibilities of your daily life			0.75 (0.03)
**MHC 11** …That you had experiences that challenged you to grow and become a better person			0.80 (0.02)
**MHC 12** …That you had warm and trusting relationships with others			0.78 (0.03)
**MHC 13** …Confident to express your own ideas and opinions			0.81 (0.02)
**MHC 14** …That your life has a sense of direction or meaning to it			0.86 (0.02)

*MHC* Mental Health Continuum

*All factor loadings are statistically significant (*p* < 0.0001)

## Discussion

In this national cross-sectional study of mixed-PGY trainees at 16 academic General Surgery Residency programs, we aimed to 1) establish validity evidence for flourishing as a measure of global wellbeing in surgical trainees, 2) assess whether evidence of validity remains regardless of gender or race, and 3) confirm that the three-factor construct underlying the MHC is supported. Our results revealed three key findings regarding General Surgery trainees: first, our data offer validity evidence for flourishing as a measure of individual global wellbeing within this population, regardless of gender or race; second, the magnitude of the effect of workplace control on MHC score differs by gender; and third, the three-factor construct of the MHC (as a measure of emotional, social and psychological wellbeing) holds.

Our first finding, that our data offer validity evidence for flourishing as a measure of individual global wellbeing in General Surgery trainees is evidenced by the positive association between flourishing and established resilience factors in surgery such as high coping skills (i.e., mindfulness) [42, 43, 47], self-efficacy (i.e., personal accomplishment) [37], and job satisfaction (i.e., perceived workplace support and control) [2, 77, 79]. This is further evidenced by the negative association between flourishing and established risk factors in surgery such as signs of distress (i.e., high stress, depression, and anxiety) [25, 30, 44, 65, 66, 79], and job strain (i.e., high perceived workplace demand) [13, 29, 42, 43]. These same relationships were reiterated across gender and race subgroups and for the total MHC score as a continuous variable.

By definition, flourishing represents the presence of high emotional, social, and psychological functioning, fundamental characteristics of the resilient phenotype as demonstrated in multiple high-stress populations [51, 69, 70, 78, 80]. There is a substantial body of literature demonstrating the clinical relevance of both trait resilience and high MHC/flourishing, linking them to reduced risk of mental illness, suicidality, burnout and healthcare utilization [42, 43, 61], thus supporting their relevance to other metrics of high interest in regard to surgeon performance, health and longevity [19, 35, 36, 61]. As such, the MHC could serve as a 14-item screen for wellbeing among individual trainees, allowing us a more nimble means of assessing individual status both cross-sectionally and longitudinally. This represents a shift, away from the current focus on trainee pathology and towards the pursuit of multi-dimensional thriving within trainees and in the field. Finally, the sensitivity of flourishing (and MHC score) to individual factors as well as workplace systems (Control and Demand) and culture (Support), suggests this measure might be useful to both assess the efficacy of interventions and to identify maladaptive workplace elements—providing guidance for resources and targets.

Our second finding, that the magnitude of the effect of workplace control on MHC score differs by gender, is evidenced by the significantly greater increase in MHC score per each unit increase in DCSQ-control score for males compared to non-males, as determined by interaction analysis. Job Demand-Resource theory suggests that job strain (which includes burnout) develops in settings where workplace demands outstrip resources [14, 56] resulting in job dissatisfaction and even pathology [49, 57, 58, 60]. A large body of empirical work shows that the negative effects of demanding work can be mitigated through increased workplace control (i.e., decision-making latitude) and support (i.e., internal resources such as coping skills, and/or external resources such as acknowledgement and appreciation) [57, 58]. In the general population, control is especially influential on job satisfaction vs strain in males [57, 58] and this relationship appears to be reiterated among male surgical trainees. This suggests that interventions aimed at understanding and increasing workplace control may prove especially beneficial to male trainee wellbeing.

Our third finding, that the three-factor construct of the MHC holds for surgical trainees, is evidenced by our confirmatory factor analysis (CFA) and sensitivity analysis (SA) which respectively show acceptable or good index fit. The latter was obtained with the removal of MHC questions 7, “How often did you feel…that people are basically good?”, and 8, “…that the way our society works made sense to you?”, which is intriguing considering the presumed prevalence of high altruism and high efficacy in surgical trainees. However, according to the MHC’s creator [32], item 7 is actually meant to reflect ‘acceptance’ and item 8 ‘social coherence’. This may explain our findings, as the ‘surgical personality’ has often been described as decisively assertive (not particularly accepting) and independent (not necessarily oriented toward being part of the group) [75]. This raises the question of how to best conceptualize social wellbeing in surgeons, suggesting value in further exploration.

Support for the MHC factor structure has been shown across cultures [31], demographics, health indicators, and time, suggesting that differences in score between groups can be attributed to the group rather than to items functioning differently. This is particularly important because it allows us to link MHC scores in surgery to risks and diagnoses established in other settings. Moreover, a multifaceted construct allows for wellbeing to be evaluated as a complex and individual experience comprising elements (and ratios of elements) that differ across individuals. As such, support for a three-factor construct means we may be able to evaluate wellbeing in spite of differences between individuals and within individuals as they change over time. This remains to be tested.

## Limitations

While our findings are promising in terms of offering validity evidence for a clinically relevant, and sensitive measure of global wellbeing in surgical trainees, they should be viewed in the context of several limitations. Our study sample size was adequate for our analyses but inadequate for performing subgroup analyses such as interaction effects by individual PGY-level and CFA by gender, race or individual PGY-level. More granular exploration of this type is essential to our comprehensive understanding of the MHC instrument and how flourishing relates to surgical training, but will require additional research. Our study population was limited to Academic General Surgery programs, which precludes capturing the differential experience of residents at Military or Community-based programs, thus limiting the generalizability of our results. Our response rate was 34% and thus possibly biased toward particularly high-performing trainees (with more discretionary time to reply) or those who are particularly distressed (with more need for an outlet). Similarly, the higher prevalence of non-male and non-white respondents in our survey may reflect over-representation of individuals with uniquely high resilience (able to overcome the adversity inherent to underrepresented groups advancing in medicine) [16] or those with uniquely high distress (due to inherent discrimination) [28]. In all these regards, reproducing our study in a comprehensive national sample would be the ideal setting to confirm and expand on our findings. Finally, our survey did not inquire about experiences with wellbeing interventions prior to residency, participants’ conceptualization of wellbeing, nor individuals current strategies to improve wellbeing. These elements were outside the scope of the present study and are reserved for future exploration.

## Conclusions

Our results offer validity evidence for flourishing, as measured by the MHC, as a metric of global wellbeing in surgical trainees and suggest that the three-factor construct of emotional, social and psychological wellbeing is supported. Thus, the MHC has promise as a measure of multifaceted wellbeing in individual trainees, as a method for identifying impactful targets, and as a means of evaluating the efficacy of wellbeing interventions.

## Data Availability

The datasets generated and analyzed during the current study are available from the corresponding author on reasonable request.
